# The relationship between emotional reactivity and adolescent non-suicidal self-injury: the mediating role of distress tolerance and gender differences

**DOI:** 10.3389/fpsyt.2026.1751873

**Published:** 2026-09-03

**Authors:** Yeqing An, Xiaonan Song, Jiayi Ou, Mei Peng

**Affiliations:** 1 Department of Teacher Education, Lyuliang University, Lyuliang, China; 2 Department of Musicology, NOVA School of Social Sciences and Humanities, NOVA University Lisbon, Lisbon, Portugal; 3 School of Mental Health, Wenzhou Medical University, Wenzhou, China

**Keywords:** adolescent, distress tolerance, emotional reactivity, gender differences, non-suicidal self-injury

## Abstract

**Background:**

Non-suicidal self-injury (NSSI) is a growing concern among adolescents, yet the emotional mechanisms underlying this behavior remain insufficiently understood. Emotional reactivity—including sensitivity, intensity, and persistence—may be associated with NSSI through its relationship with adolescents’ capacity to tolerate emotional distress, and this indirect association may differ by gender.

**Methods:**

A total of 3,640 adolescents (M_age_ = 18.27, SD = 1.45; 52.34% were male) completed measures of emotional reactivity, distress tolerance, and NSSI behaviors. Structural equation modeling was used to examine the mediating role of distress tolerance in the link between emotional reactivity and NSSI, and multi-group analyses were conducted to explore gender-related patterns.

**Results:**

Emotional reactivity was significantly and positively associated with NSSI. Distress tolerance mediated the association between emotional reactivity and NSSI: higher emotional reactivity was associated with lower distress tolerance, which in turn was associated with higher levels of NSSI. Gender-stratified analyses showed that the indirect effects were significant among females but not males. Further examination of the dimensions of emotional reactivity revealed that gender differences were significant for emotional intensity but not for emotional sensitivity or emotional persistence.

**Conclusions:**

These findings suggest that distress tolerance is a key psychological factor underlying the association between emotional reactivity and NSSI, with this association being particularly salient among female participants. Prevention and intervention programs should focus on strengthening distress-tolerance skills and providing gender-sensitive support to reduce NSSI risk among adolescents.

## 1 Introduction

In recent years, the prevalence of non-suicidal self-injury (NSSI) among adolescents has continued to rise, becoming a major public health concern worldwide (1). Empirical evidence indicates that the prevalence of NSSI among adolescents ranges from approximately 17.7% to 25% (2, 3), with a lifetime prevalence of 14.5% (4). NSSI refers to direct, deliberate, and repetitive damage to adolescents’ own body tissue without suicidal intent and without social or cultural sanction. Common forms include cutting, burning, stabbing, biting, hitting, and excessive rubbing (5, 6). Beyond the direct physical harm it causes, NSSI is strongly associated with anxiety, depression, and increased suicide risk among adolescents (7, 8). Notably, NSSI is regarded as a significant predictor of future suicidal behaviors (9). Compared with other age groups, adolescents experience heightened emotional instability and more intense negative emotions; some may engage in self-injury to alleviate psychological distress (10, 11). Furthermore, NSSI is closely linked to current and long-term impairments in physical health, emotion regulation, and interpersonal functioning (12, 13). Therefore, systematically examining the factors and underlying mechanisms contributing to adolescent NSSI is of considerable theoretical and practical significance for promoting adolescent mental health and preventing extreme behaviors.

Previous research has primarily focused on external or comorbid factors—such as depression, anxiety, early family environment, and peer relationships—while relatively few studies have examined the role of core emotional processes in NSSI (14). According to the functional model of experiential avoidance, heightened emotional intensity and low frustration tolerance are critical risk factors for adopting experiential avoidance strategies (e.g., NSSI) to manage overwhelming emotions. Existing findings provide preliminary support for this possibility: adolescents who engage in NSSI tend to interpret neutral facial expressions (e.g., happy or disgusted) as more threatening and display heightened emotional reactivity to negative social cues (15). Although prior research has touched on certain dimensions of emotional responding, an integrated examination of multidimensional emotional reactivity remains insufficient. Building on previous findings, the present study conceptualizes negative emotional reactivity across three dimensions—emotional sensitivity, emotional intensity, and emotional persistence—and aims to examine its association with NSSI and the underlying psychological mechanisms. This approach seeks to offer a developmental perspective that may inform theories and interventions on stress coping and emotion regulation among adolescents.

Emotional reactivity refers to the degree of sensitivity to emotional stimuli, the magnitude of emotional experiences, and the duration for which emotional states are maintained (16). Prior studies have shown that adolescents with heightened emotional reactivity are more likely to experience intense emotional fluctuations when confronted with stress or negative affect and encounter greater difficulty returning to baseline emotional states. Such patterns increase the likelihood of impulsive behaviors and self-injury (17). According to the experiential avoidance model, NSSI may serve as a coping strategy whereby adolescents attempt to rapidly alleviate or escape from intolerable negative emotions (18). When external stressors—such as academic pressure or interpersonal conflict—evoke anger, frustration, or self-blame, adolescents who experience relief or emotional numbing after engaging in NSSI may gradually rely on this behavior as a maladaptive regulation strategy, forming a conditioned association between heightened arousal and self-injury (19). From an ecological perspective, NSSI is shaped by both adolescent characteristics (e.g., emotional instability, alexithymia, cognitive rigidity) and environmental factors (e.g., dysfunctional family dynamics, school bullying, peer rejection) (20, 21). Adolescents with high emotional reactivity are more likely to internalize unexpressed emotions and externalize psychological pain into physical injury when emotional load exceeds tolerance (22). In addition, emotional instability may limit cognitive flexibility, increasing rigid self-injurious ideation and elevating the frequency and severity of NSSI (23). Based on this evidence, we propose Hypothesis 1 (H1): Emotional reactivity is positively associated with adolescents’ NSSI behaviors.

Although emotional reactivity is associated with the risk of NSSI, its underlying mechanisms remain unclear. Distress tolerance, a key emotion regulation variable, warrants particular attention. According to the integrated model of NSSI, self-injury functions both as an emotional and cognitive regulatory strategy and as a means of communicating internal states or influencing others (18, 24). Genetic predispositions, childhood trauma, family environment, and parenting practices are associated with adolescents’ emotional perception, regulation, and tolerance, as well as with difficulties in emotion management, interpersonal problems, and self-injurious behavior (25, 26). Distress tolerance refers to the capacity to withstand negative emotional states while maintaining functioning. It comprises several dimensions, including tolerance of uncertainty, physical discomfort, ambiguous situations, frustration, and negative affect (27). Prior research suggests that emotional reactivity is associated with the level of distress tolerance among adolescents when facing negative emotions, with those higher in emotional reactivity generally exhibiting lower distress tolerance (28). Reduced tolerance may foster maladaptive coping behaviors; adolescents with low distress tolerance are more inclined to adopt rapid-relief strategies, such as NSSI, to escape overwhelming emotional experiences (29–31). A review by Akbari et al. (29) further indicates that distress tolerance functions as a risk factor for NSSI and may represent a transdiagnostic protective factor. These findings suggest that lower distress tolerance may help explain the association between emotional reactivity and NSSI. Therefore, we propose Hypothesis 2 (H2): Distress tolerance mediates the association between emotional reactivity and adolescent NSSI.

Moreover, previous research has documented significant gender differences in emotional processing and NSSI (32). As a relatively stable individual characteristic that is less susceptible to situational fluctuations, gender is often examined as a moderator (33). Although contextual factors may influence emotional responses and NSSI, these associations may differ by gender. Existing findings regarding the association between emotional reactivity and NSSI are inconsistent and can be broadly grouped into three patterns. One group of studies conducted with adolescent samples found that the association between emotional reactivity and NSSI was significantly stronger among females than among males, indicating a significant moderating effect of gender. A second group of studies found no significant gender differences, suggesting that the positive association between emotional reactivity and NSSI was comparable among male and female adolescents. A third, smaller group of studies focusing on late-adolescent male samples found a stronger association between high emotional reactivity and impulsive forms of NSSI among males. These inconsistent findings may be attributable to differences in sample age ranges, regional gender norms, and other potential confounding factors.

Similarly, findings regarding gender differences in the prevalence of NSSI remain mixed, and three main perspectives have emerged in the literature. The first is the male vulnerability perspective, which proposes that males face greater family expectations and more stringent external constraints and may therefore be more likely to use NSSI to release emotional pressure after prolonged emotional suppression. The second is the female vulnerability perspective, which suggests that females tend to be more emotionally sensitive, have lower tolerance for emotional distress, and are consequently more likely to engage in NSSI. The third is the no-difference perspective, which argues that male and female adolescents do not differ substantially in emotional susceptibility and that overall NSSI risk does not differ significantly between males and females. Although gender differences in the prevalence of NSSI remain debated, numerous studies have identified gender-specific patterns in the methods of self-injury: females more frequently engage in cutting and scratching, whereas males more often engage in hitting, burning, or skin-picking (34). According to gender socialization theory, societal expectations shape emotional expression and behavioral responses. Females are encouraged to express emotions and thus are more susceptible to internalizing problems (e.g., depression, suicidal ideation), whereas males are socialized to suppress emotions and are more likely to display externalizing behaviors (e.g., aggression, substance use) (35). To date, few studies have directly examined gender differences in the association between emotional reactivity and NSSI among adolescents, and existing findings remain inconsistent. Understanding the mechanisms through which emotional reactivity contributes to NSSI requires incorporating gender into the analytical framework. Drawing on gender socialization theory and gender-related differences in emotional processing during adolescence, the present study hypothesizes that the positive association between emotional reactivity and NSSI will be stronger among female adolescents than among male adolescents.

Therefore, we propose Hypothesis 3 (H3): Gender moderates the association between emotional reactivity and NSSI, such that the positive association is stronger among female adolescents than among male adolescents.

In summary, guided by the integrated model of NSSI and the experiential avoidance framework, this study develops a mediation model to examine the association between emotional reactivity and adolescent NSSI and explore the potential underlying pathways.

## 2 Methods

### 2.1 Ethical approval

This study was approved by the Ethics Committee of Lyuliang University. Written informed consent was obtained from schools, parents or legal guardians, and all participating students. All assessors received standardized training prior to data collection to ensure consistent instructions and procedures. Participants were assured that their responses would remain confidential, used solely for research purposes, and securely stored. They were also informed that participation was voluntary and that they could withdraw at any time without penalty.

### 2.2 Participants

In December 2021, adolescents were recruited through convenience sampling from Shanxi Province, the Inner Mongolia Autonomous Region, and several other regions of China. A total of 4,100 adolescents participated in the survey. All procedures involving human participants were conducted in accordance with the ethical principles of the Declaration of Helsinki and its subsequent amendments. With the cooperation and support of school administrators, students read the survey instructions and completed written informed consent forms in their classrooms before completing the online questionnaire using school-provided electronic devices.

Clear eligibility criteria and questionnaire validity criteria were established. The inclusion criteria were as follows: (a) being an enrolled student aged 10–19 years; (b) provision of written informed consent by both the participant and a parent or legal guardian; and (c) the ability to complete the online questionnaire independently. The exclusion criteria were: (a) a diagnosis of severe mental illness; (b) cognitive impairment; and (c) exposure to a major traumatic event within the previous three months. Questionnaires were considered invalid if more than 20% of the items were unanswered, if the same response option was selected consecutively across multiple items, or if the questionnaire was completed in less than 3 minutes. Following data collection, all responses were systematically screened. After responses with missing information or evidence of careless or inattentive responding were excluded, 3,640 valid questionnaires were retained, representing 88.78% of the original sample.

Consistent with the World Health Organization’s definition of adolescence, participants were aged 10–19 years. Their mean age was 18.27 years (SD = 1.45); 52.34% were male, 26.87% were only children, and 53.85% were in their first or second year of college. Participants rated the quality of their parents’ marital relationship on a 5-point scale. The mean score was 1.09 ± 0.29, suggesting that most participants perceived their parents’ relationship as relatively harmonious. Information was also collected on parental survival status, educational attainment, and occupation to characterize participants’ family backgrounds and socioeconomic circumstances. Detailed sample characteristics are presented in **Table 1**. These demographic and family variables were subsequently included as covariates to adjust for potential confounding effects of individual- and family-level factors on adolescent NSSI.

**Table 1:** Demographic characteristics of the sample (N = 3,640).

Variable	Category	n(%)/M ± SD
Age(years, M ± SD)		18.27 ± 1.45
Gender	Male	1905(52.34%)
	Female	1735(47.66%)
Educational stage	Junior high school	249(6.84%)
	Senior high school	173(4.75%)
	Junior college	1258(34.56%)
	Undergraduate	1960(53.85%)
Only Child	Yes	978(26.87%)
	No	2662(73.13%)
Mother Alive	Yes	3599(98.87%)
	No	41(1.13%)
Father Alive	Yes	3545(97.39%)
	No	95(2.61%)
Mother’s Education	College or above	282(7.75%)
	Technical secondary school	736(20.22%)
	Junior high school	828(22.75%)
	Primary school or below	1794(49.28%)
Mother’s Occupation	Worker	684(18.79%)
	Farmer	2017(55.41%)
	Professional	150(4.12%)
	Cadre	74(2.03%)
	Other	715(19.64%)
Father’s Education	College or above	338(9.29%)
	Technical secondary school	864(23.73%)
	Junior high school	1829(50.25%)
	Primary school or below	609(16.73%)
Father’s Occupation	Worker	1116(30.66%)
	Farmer	1819(49.97%)
	Professional	98(2.69%)
	Cadre	147(4.04%)
	Other	460(12.64%)
Parental Relationship Quality(M ± SD)		1.09 ± 0.29

Except for age and parental relationship quality (presented as M ± SD), all values are presented as n (%).

A risk assessment was also conducted for participants who reported NSSI during data collection. Participants who reported recurrent NSSI, defined as five or more episodes during the previous year, or current, intense suicidal ideation were provided with standardized information about psychological support, including contact information for the school counseling center and a free mental health helpline. They were also encouraged to seek professional assistance to reduce potential psychological risk.

### 2.3 Measures

#### 2.3.1 Adolescent self-harm scale

Adolescents’ NSSI behaviors were assessed using the Adolescent Self-Harm Scale (ASHS) (36). The scale consists of 19 items, including 18 closed-ended and one open-ended item. The frequency of self-harm behaviors was assessed on a four-level scale (0 = 0 times, 1 = once, 2 = 2–4 times, 3 = ≥5 times). The severity of physical injury was rated on a five-level scale (0 = none, 1 = mild, 2 = moderate, 3 = high, 4 = extremely severe). For each of the 18 closed-ended items, the frequency score was multiplied by the corresponding severity score. The 18 item scores were then summed to obtain the total NSSI score, with higher scores indicating more severe NSSI. In this study, the internal consistency of the scale was excellent (Cronbach’s α = 0.96).

#### 2.3.2 Emotion reactivity scale

The Emotion Reactivity Scale (ERS) was used to measure adolescents’ emotional reactivity (16). The ERS comprises 21 items across three dimensions: emotional sensitivity (10 items), emotional intensity (7 items), and emotional persistence (4 items). Each dimension was scored separately. Responses were rated on a 5-point scale (0 = “not at all like me” to 4 = “completely like me”), with higher scores indicating greater emotional reactivity. The internal consistency of the scale in the present study was high (Cronbach’s α = 0.957).

#### 2.3.3 Distress tolerance scale

Adolescents’ distress tolerance was assessed using the Distress Tolerance Scale (DTS) (37). The scale contains 15 items rated on a 5-point scale (1 = “strongly agree” to 5 = “strongly disagree”), with one item (Item 6) positively worded to reflect higher distress tolerance, which was reverse-scored prior to data analysis. Total scores were calculated by summing all item responses, with possible scores ranging from 15 to 75. Higher total scores indicate higher levels of distress tolerance. In this study, the internal consistency of the scale in this sample was satisfactory (Cronbach’s α = 0.932).

### 2.4 Procedure and data analysis

Prior to formal data collection, all test administrators received standardized training to ensure consistency in the instructions and administration procedures and to enable participants to understand the study information clearly and accurately. After permission had been obtained from the participating schools and informed consent had been obtained from the participants and their parents or legal guardians, trained school mental health teachers and undergraduate research assistants served as test administrators, while homeroom or subject teachers provided on-site assistance. Each classroom was supervised by at least one test administrator and one assisting teacher. Written informed consent forms were distributed to all participants. After reading the study information and providing informed consent, students completed the online questionnaire using school-provided electronic devices in their classrooms. Test administrators answered participants’ questions during the survey, and students submitted their responses online immediately after completing the questionnaire. The questionnaire took approximately 10 minutes to complete.

Because questions concerning NSSI and emotional functioning involve sensitive psychological topics, administering the questionnaire collectively in classroom settings may have increased the likelihood of socially desirable responding. The following measures were therefore implemented to reduce this potential bias. First, the survey was conducted anonymously, and no personally identifiable information, such as participants’ names or class information, was collected. Before the survey, participants were explicitly informed that their teachers and school administrators would not have access to their individual responses and that the data would be used solely for research purposes. Second, the standardized instructions emphasized that there were no right or wrong answers and that the purpose of the survey was to obtain an accurate understanding of adolescents’ psychological experiences. Third, students were seated apart and completed the questionnaire independently, while homeroom and subject teachers were responsible only for maintaining order in the classroom. Fourth, attention-check items and reverse-scored items were included in the questionnaire. Questionnaire completion times were also examined, and responses showing evidence of careless or random responding or excessively short completion times were excluded to reduce the influence of invalid responding.

Descriptive statistics and correlation analyses were conducted using SPSS 27.0. The dimensions of emotional reactivity were treated as the independent variables, distress tolerance as the mediating variable, and the total NSSI score as the dependent variable. These variables were entered into the model as observed variables. Observed-variable path analysis was conducted using Mplus version 8.3 to simultaneously test the hypothesized mediation pathways. The total-effect model was tested first, followed by the mediation model in which distress tolerance was specified as the mediator. Bias-corrected bootstrapping with 5,000 resamples was used to estimate the 95% confidence intervals for the indirect effects. An indirect effect was considered statistically significant when its confidence interval did not include zero. The κ² index was also calculated to quantify the magnitude of each indirect effect.

To examine whether the mediation pathways differed between male and female participants, multi-group path analysis was conducted. The mediation model was first estimated separately for the male and female groups to evaluate model fit in each group. An unconstrained multi-group model, in which all structural path coefficients were freely estimated across groups, was then compared with a constrained model in which the corresponding structural path coefficients were set equal across groups. A chi-square difference test was used to determine whether the structural paths differed overall between the two groups. If the overall test was statistically significant, additional parameter-difference tests were conducted to identify the specific paths that differed between male and female participants.

## 3 Results

### 3.1 Common method bias

Given that all variables were assessed using self-report measures, Harman’s single-factor test was conducted to examine potential common method bias (38). Ten factors with eigenvalues greater than 1 were extracted, and the first factor accounted for 20.08% of the total variance, which was below the critical threshold of 40%. These results indicated that common method bias was not a serious concern in the present study.

### 3.2 Descriptive statistics and correlations

Descriptive statistics and correlation coefficients are presented in **Table 2**. Based on the scoring and classification criteria of the Adolescent Self-Harm Scale, the score for each item was calculated by multiplying the frequency of self-injury by the degree of harm, and the total scale score was obtained by summing across all items. A total score > 0 was defined as the presence of non-suicidal self-injury (NSSI) behaviors, while a total score = 0 was defined as the absence of NSSI behaviors. According to this criterion, among the 3,640 valid samples in this study, 388 participants exhibited NSSI behaviors, yielding an overall NSSI detection rate of 10.66%. In addition, referring to the DSM-5 diagnostic criteria for NSSI, a total frequency of self-injury of ≥ 5 times within the past year was used to define the clinically significant recurrent NSSI subgroup. In this study, 146 participants (4.01% of the total sample) were identified as exhibiting recurrent NSSI.

**Table 2:** Descriptive statistics and correlation matrix of all variables (N = 3640).

Variable	1	2	3	4	5	6	7	8	9	10	11	12	13	14	15
1 Gender	1														
2 Educational Stage	0.03	1													
3 Only Child	-0.09^**^	0.21^**^	1												
4 Mother Alive	-0.02	0.02	-0.02	1											
5 Father Alive	-0.07^**^	0.01	0.01	0.13^**^	1										
6 ME	-0.02	0.06^**^	0.27^**^	0.02	0.03^*^	1									
7 MO	-0.08^**^	-0.10^**^	-0.05^**^	0.05^**^	-0.01	-0.16^**^	1								
8 FE	-0.02	0.06^**^	0.22^**^	0.02	0.04^*^	0.55^**^	-0.15^**^	1							
9 FO	-0.09^**^	-0.09^**^	-0.02	-0.01	0.07^**^	-0.15^**^	0.52^**^	-0.13^**^	1						
10 PRQ	-0.04^**^	-0.02	-0.10^**^	0.06^**^	0.05^**^	-0.01	0.05^**^	0.01	0.04^**^	1					
11 EI	-0.10^**^	-0.15^**^	-0.01	0.03	0.01	-0.01	0.06^**^	-0.01	0.03	0.17^**^	1				
12 EP	-0.13^**^	-0.11^**^	0.02	0.02	0.01	0.01	0.04^*^	0.02	0.02	0.14^**^	0.78^**^	1			
13 ES	-0.14^**^	-0.13^**^	-0.01	0.02	0.01	-0.01	0.06^**^	-0.01	0.03	0.18^**^	0.88^**^	0.84^**^	1		
14 DT	0.06^**^	0.13^**^	0.01	0.01	0.01	-0.02	-0.01	0.01	-0.02	-0.08^**^	-0.43^**^	-0.44^**^	-0.47^**^	1	
15 NSSI	-0.03	-0.08^**^	0.01	0.02	-0.01	-0.01	0.01	-0.04^*^	0.02	0.10^**^	0.27^**^	0.24^**^	0.27^**^	-0.17^**^	1
*M*	1.52	3.36	1.73	1.01	1.03	2.87	2.48	2.74	2.18	1.1	5.36	3.75	7.56	52.68	0.54
*SD*	0.5	0.86	0.44	0.11	0.16	0.85	1.36	0.84	1.27	0.29	4.85	3.2	7.27	12.35	2.37

ME, Mother’s Education; MO, Mother’s Occupation; FE, Father’s Education; FO, Father’s Occupation; PRQ, Parental Relationship Quality; EI, Emotional Intensity; EP, Emotional Persistence; ES, Emotional Sensitivity; DT, Distress Tolerance; NSSI, Non-suicidal Self-injury. ^*^p < 0.05, ^**^p < 0.01, ^***^p < 0.001.

Emotional sensitivity, emotional intensity, and emotional persistence were all positively correlated with NSSI behaviors. All three dimensions of emotional reactivity were significantly negatively correlated with distress tolerance. Distress tolerance showed a moderate negative correlation with adolescents’ NSSI behaviors.

### 3.3 Mediation model testing: emotional reactivity and NSSI

We first tested the total effect of emotional reactivity on adolescent NSSI using observed-variable path analysis. The model demonstrated good fit (χ^2^/df = 7.13, RMSEA = 0.04, CFI = 0.93, TLI = 0.92, SRMR = 0.04). Emotional intensity significantly predicted NSSI (β = 0.15, *p* < 0.001), whereas the total effects of emotional persistence (β = 0.04, *p* > 0.05) and emotional sensitivity (β = 0.04, *p* > 0.05) were nonsignificant.

**Figure 1:**
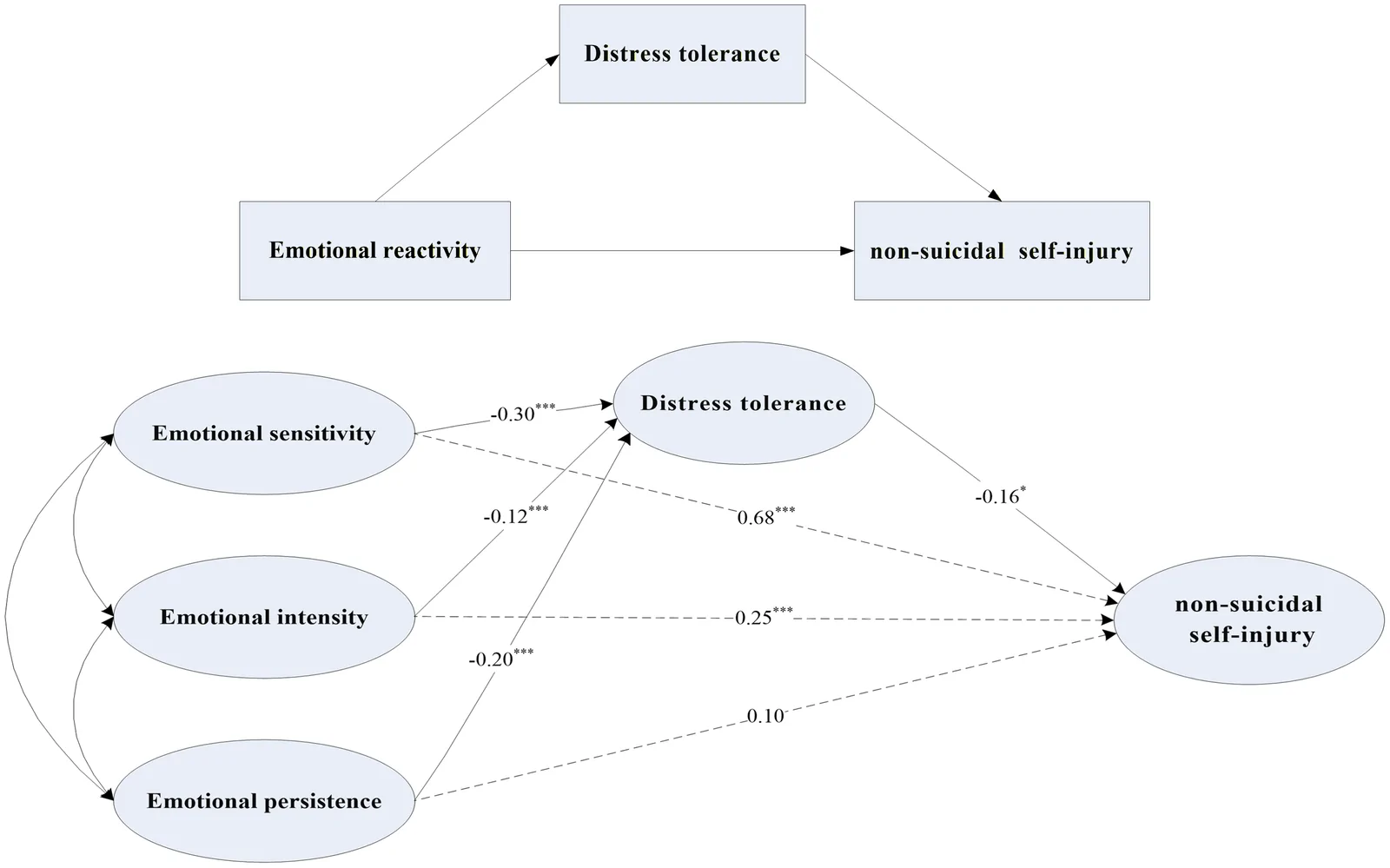
Mediation effect of distress tolerance on the relationship between emotional reactivity and non-suicidal self-injury. Control variables (adolescent gender, educational stage, mother’s occupation, and parental relationship quality) were included in the model. *p < 0.05, ***p < 0.001.

Next, distress tolerance was added as a mediator. The mediation model also showed satisfactory fit (χ^2^/df = 5.41, RMSEA = 0.04, CFI = 0.94, TLI = 0.93, SRMR = 0.04). As shown in **Figure 1**, emotional sensitivity, emotional intensity, and emotional persistence each significantly negatively predicted distress tolerance (β = –0.30, β = –0.12, β = –0.20, respectively; all *p* < 0.001). Distress tolerance significantly negatively predicted adolescents’ NSSI behaviors (β = –0.16, *p* < 0.05).

Bootstrapping with bias correction was further conducted to examine the significance of indirect effects, and *κ*^2^ effect size values were calculated. As shown in **Table 3**, the 95% confidence intervals of the indirect effects for all three dimensions of emotional reactivity did not include zero. The effect sizes (*κ*^2^) for emotional sensitivity and emotional intensity were both below 0.09, indicating small effect sizes. The indirect effect of emotional persistence yielded a *κ*^2^ value greater than 0.09, representing a medium effect size.

**Table 3:** 95% Confidence intervals and effect sizes for the mediation effects.

Mediation paths	Total sample			Males			Females		
	Estimate	95%CI	*κ* ^2^	Estimate	95%CI	*κ* ^2^	Estimate	95%CI	*κ* ^2^
Emotional Sensitivity → Distress Tolerance → Non-Suicidal Self-Injury	0.05	[0.02,0.10]	0.07	0.01	[-0.01,0.04]	0.04	0.05	[0.02,0.11]	0.08
Emotional Intensity → Distress Tolerance → Non-Suicidal Self-Injury	0.02	[0.002,0.04]	0.07	0.00	[-0.00,0.02]	0.07	ns	[0.00,0.06]	0.08
Emotional Persistence → Distress Tolerance → Non-Suicidal Self-Injury	0.03	[0.01,0.06]	0.24	0.01	[-0.01,0.03]	0.16	ns	[0.01,0.10]	0.41

### 3.4 Gender differences

We first tested the mediation model separately for male and female adolescents to determine whether multi-group path analysis could be applied. The model demonstrated good fit for both groups (Males: χ²/df = 5.45, RMSEA = 0.05, CFI = 0.93, TLI = 0.92, SRMR = 0.05; Females: χ²/df = 4.27, RMSEA = 0.04, CFI = 0.91, TLI = 0.91, SRMR = 0.04).

**Figure 2:**
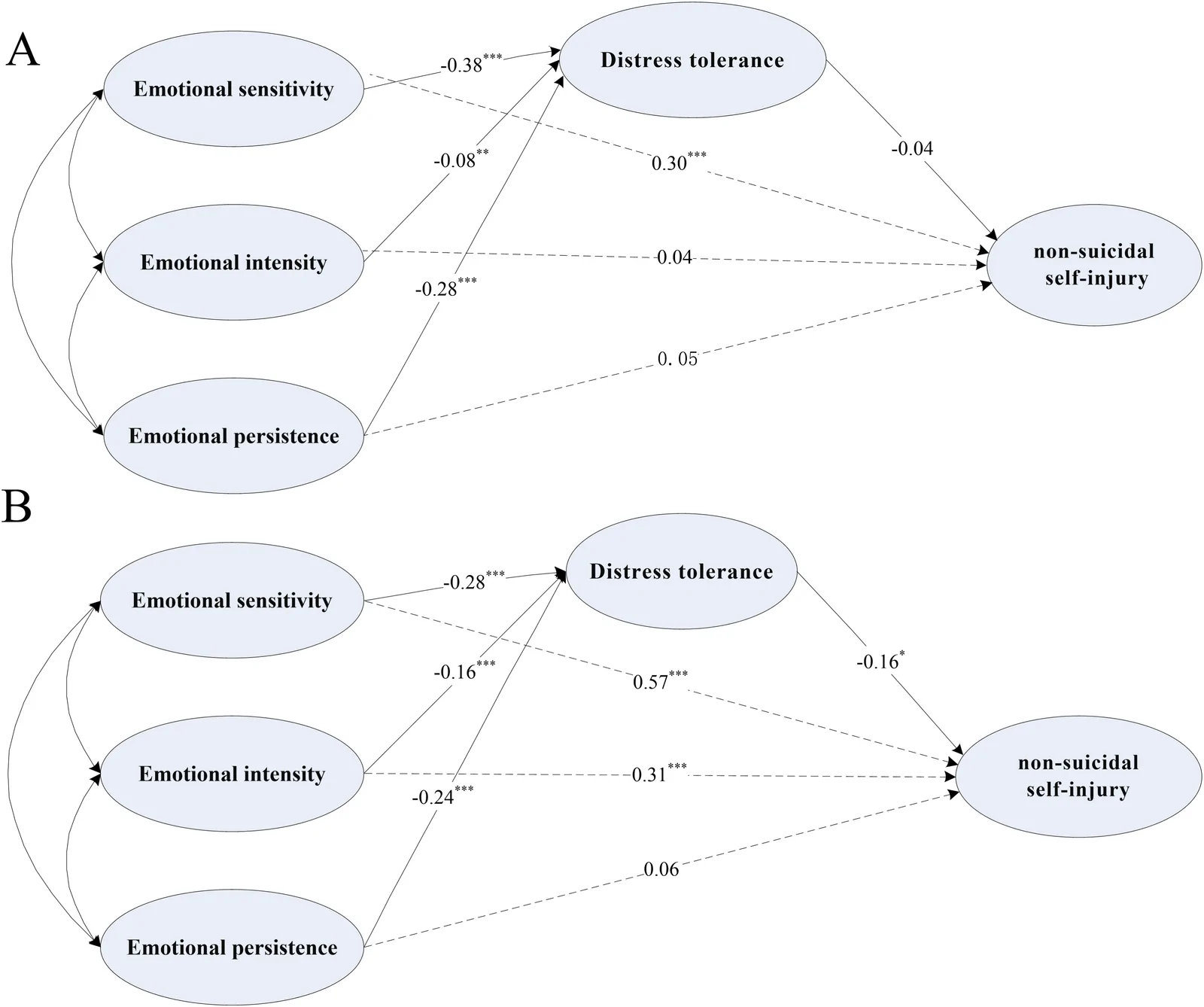
Multi-group comparisons of emotional reactivity, distress tolerance, and non-suicidal self-injury. Control variables (adolescent educational stage, mother’s occupation, and parental relationship quality) were included in the model. **(A)** shows results for males, and **(B)** shows results for females. *p < 0.05, **p < 0.01, ***p < 0.001.

In Model 1, all structural path coefficients were freely estimated across gender groups. Model fit remained satisfactory (χ²/df = 5.55, RMSEA = 0.04, CFI = 0.93, TLI = 0.92, SRMR = 0.05). In Model 2, the corresponding structural path coefficients were constrained to be equal across gender groups, and again showed good fit (χ²/df = 5.70, RMSEA = 0.04, CFI = 0.93, TLI = 0.92, SRMR = 0.05). A chi-square difference test revealed a significant difference between the two models [Δχ² (38) = 56.3, *p* < 0.05], indicating significant gender differences (see **Figure 2**).

Distress tolerance showed a significant negative association with NSSI among females (β_female = –0.16, *p* < 0.05), but not among males (β_male = –0.04, *p* > 0.05); however, the difference between the two coefficients was nonsignificant (c.r. = –0.15, *p* > 0.05). Emotional sensitivity significantly predicted distress tolerance in both groups (β_male = –0.38, β_female = –0.28; both *p* < 0.001), with no significant difference (c.r. = –0.30, *p* > 0.05). Emotional persistence also significantly predicted distress tolerance (β_male = –0.24, β_female = –0.28; both *p* < 0.001), and this difference was nonsignificant (c.r. = –0.24, *p* > 0.05). Emotional intensity significantly predicted distress tolerance as well (β_male = –0.08, *p* < 0.01; β_female = –0.16, *p* < 0.001), with no significant group difference (c.r. = –0.30, *p* > 0.05).

Bootstrapping results further showed that, among males, the 95% confidence intervals for the indirect effects of emotional sensitivity, emotional intensity, and emotional persistence all included zero, indicating nonsignificant mediation. In contrast, among females, the 95% confidence intervals for all three indirect effects did not include zero. The *κ*^2^ values for emotional sensitivity and emotional intensity were below 0.09 (small effects), whereas the *κ*^2^ for emotional persistence exceeded 0.09 (medium effect).

## 4 Discussion

In the new era of mental health research, NSSI has drawn increasing public and academic attention. From the perspective of adolescent emotional differences, this study integrates the integrated model of NSSI and the experiential avoidance model, constructing a theoretical framework in which distress tolerance functions as a mediator and gender serves as a moderator. This framework not only identifies an indirect pathway through which emotional reactivity is associated with NSSI via distress tolerance, but also further illuminates how this association varies across genders. The findings provide important theoretical insights into the mechanisms linking emotional reactivity and adolescent NSSI and offer guidance for developing gender-sensitive prevention and intervention strategies.

### 4.1 The relationship between emotional reactivity and adolescent NSSI

This study found that adolescents’ emotional reactivity was significantly and positively associated with NSSI, with emotional intensity showing the most stable direct association. This finding aligns with previous research suggesting that adolescents with heightened emotional responses are more likely to engage in NSSI. Specifically, adolescents characterized by high negative emotional intensity, sustained attention to negative stimuli, and prolonged emotional states are more prone to self-injurious behaviors (39, 40).

The results support the biosocial model of self-injury (41), which posits that early contextual factors exert long-term influences on self-injurious behavior. Psychological deficits and stress exposure measured between ages 8 and 12 can predict girls’ NSSI during early adolescence and even into early adulthood (42). This pattern is also consistent with ecological systems theory, as adolescents transitioning to university face increasing social demands, complex interpersonal challenges, and rapidly changing environments in which emotional processes play a crucial role (43). In addition, within the broader context of the digital age, exposure to cyberbullying and misinformation may heighten anger and emotional volatility, thereby increasing the likelihood of impulsive self-injury (14).

Furthermore, the present study extends existing literature by identifying emotional sensitivity, emotional intensity, and emotional persistence as significant predictors of NSSI. These findings suggest that both long-term accumulation of negative emotions and heightened reactivity to negative information may be associated with maladaptive coping behaviors. Strengthening emotional guidance and providing early intervention may therefore help adolescents develop healthier emotion management capacities and reduce NSSI risk.

### 4.2 The mediating role of distress tolerance

The results revealed a significant indirect association between emotional reactivity and NSSI through distress tolerance. This finding broadens the theoretical scope of emotional reactivity and supports a central tenet of the experiential avoidance model: adolescent’ individual characteristics (e.g., emotional reactivity) are closely associated with their capacity to withstand and cope with emotional distress, and together they are associated with adaptive functioning and maladaptive behaviors (44).

Adolescents high in emotional reactivity are associated with more intense emotional pain and avoidance motivation when confronted with negative stimuli. Non-suicidal self-injury (NSSI) is closely associated with such discomfort and is often employed as a coping strategy by these adolescents (45). Although NSSI co-occurs with short-term emotional relief (1), in the long run, NSSI, emotional avoidance patterns, and tendencies toward emotional deterioration tend to intertwine, while lower distress tolerance and poorer psychological adjustment frequently co-occur as well (46, 47).

Moreover, the associations of emotional sensitivity and emotional persistence with NSSI appear to operate primarily through distress tolerance rather than through direct pathways. Heightened emotional reactivity is associated with lower distress tolerance, which in turn is associated with greater susceptibility to NSSI. Distress tolerance may therefore serve as an important link between emotional processes and behavioral outcomes.

From an applied perspective, although distress tolerance is relatively difficult to modify, emotional reactivity is more amenable to intervention through cognitive-behavioral therapy, dialectical behavior therapy, and emotion-regulation group interventions (15, 48). Therefore, educators and mental health practitioners may focus on improving adolescents’ emotional awareness, regulation skills, and cognitive coping strategies to interrupt maladaptive cycles and promote emotional and psychological resilience. These findings also highlight those clinical interventions should prioritize improving adolescents’ tolerance of emotional distress, rather than solely targeting emotional reactivity itself.

### 4.3 Gender differences

The present study found no significant gender differences in the overall prevalence of adolescent NSSI, a result that contrasts with some prior findings. Existing literature generally falls into three perspectives. The “male vulnerability” perspective argues that males experience harsher punishment, greater familial expectations, and heavier psychological burdens, and that personality characteristics such as rigidity may increase their likelihood of engaging in extreme behaviors such as NSSI (49–51). The “female vulnerability” perspective posits that females’ heightened emotional sensitivity, richer emotional experiences, limited access to adaptive emotion-regulation strategies, and greater difficulty controlling impulses may elevate their risk for NSSI (52, 53). The “no difference” perspective suggests that males and females do not differ substantially in emotional susceptibility (54), a view supported by the present study.

These discrepancies may reflect differences in research methodologies, measurement tools, sampling contexts, and cultural backgrounds. They may also derive from evolving gender roles and greater freedom in emotional expression, which may be weakening the influence of traditional gender norms on emotional and behavioral patterns. According to the biosocial model (41), high emotional reactivity involves heightened sensitivity and intense emotional responses. Males may adopt a “high accumulation–high eruption” pattern due to long-term emotional suppression, whereas females may display a “high frequency–cyclical relief” pattern owing to relatively greater emotional expressiveness. Although the triggers and manifestations of NSSI may differ by gender, the core functions of NSSI—such as emotional relief and attentional redirection—tend to be consistent across genders (55). Once the “emotional relief–behavior reinforcement” loop is formed, the maintenance of NSSI may transcend gender (56).

Importantly, although the overall prevalence of NSSI was comparable between male and female adolescents, the strength of the association between emotional reactivity and NSSI differed between the two groups, providing empirical support for Hypothesis 3. Among females, distress tolerance played a central mediating role: heightened emotional reactivity co-occurred with lower distress tolerance, alongside a greater reliance on NSSI as a coping strategy. In contrast, among males, the association between emotional reactivity and NSSI appeared to be driven by other mechanisms, such as impulsivity, externalizing tendencies, or substance use (57). These differences may reflect distinct emotion-regulation patterns shaped through gender socialization. Females may be more likely to internalize emotional distress, often following a “tolerance–accumulation–release” pattern in which NSSI provides temporary relief (58). Conversely, males may be more likely to externalize distress through aggression or impulsive reactions, a pattern that may be associated with a weaker mediating role of distress tolerance and stronger pathways involving externalizing behaviors (59).

However, whether distress tolerance itself exhibits stable gender differences remains unclear (60). Research on NSSI has historically focused more heavily on female samples, resulting in limited understanding of distress tolerance in males. Some cross-study evidence shows that males generally exhibit higher levels of aggression (61), suggesting the need to consider links between NSSI and externalizing characteristics—such as impulsivity, aggression, and inhibitory control difficulties—when studying male adolescents.

These findings highlight the importance of gender-sensitive practices in intervention. Effective prevention should account for gender-specific emotional and behavioral patterns, targeting the reinforcement cycles that maintain NSSI and promoting adolescents’ overall emotional health and behavioral competence.

### 4.4 Implications and limitations

This study has several implications for the prevention of and intervention for adolescent NSSI. Parents should pay attention to their children’s emotional states, provide timely support when they experience negative emotions, and avoid prolonged conflicts that may lead adolescents to use self-injury as a means of coping with unresolved internal distress. Schools and communities may develop psychological intervention programs aimed at enhancing distress tolerance in response to the widespread stressors faced by adolescents, thereby promoting emotional adjustment and overall well-being (19).

This study also has several limitations. First, although the research model was theoretically grounded, the cross-sectional design means that the findings can only reflect associations among the variables and cannot support causal inferences. Future research could employ longitudinal designs to further examine the long-term association between emotional reactivity and nonsuicidal self-injury (NSSI), as well as the underlying pathways. In addition, this study primarily used adolescent self-report measures to assess the relevant variables. Although self-reports can effectively capture adolescents’ own experiences and psychological states, reliance on a single source of information may introduce measurement bias. Future studies could incorporate multiple informants, such as parent and teacher ratings, to assess the study variables from different perspectives, thereby enhancing the objectivity of the measurements and the reliability of the conclusions.

Second, guided by the experiential avoidance model, this study focused specifically on examining the mediating role of distress tolerance. Future research could simultaneously include variables such as emotion regulation difficulties to compare the relative contributions of different psychological mechanisms in the development of NSSI. Moreover, this study only explored gender differences; future investigations could incorporate family and social factors—such as only-child status and parental relationship quality—to further reveal the interactive effects of individual characteristics and environmental variables on NSSI. In addition, future studies could consider the influence of regional cultural characteristics and differences in school educational resources on adolescent NSSI, thereby enhancing the generalizability of the findings.

Third, the data in this study were collected online in classroom group settings. Sensitive indicators such as NSSI and negative emotions may be susceptible to social desirability bias. Future studies could consider administering the measures individually to reduce response interference caused by the external environment.

Fourth, the sample had an uneven age and educational distribution. The participants in this study were predominantly aged 16-19, with relatively few adolescents aged 10-15. Therefore, the conclusions regarding NSSI and emotion regulation cannot be fully generalized to younger adolescent populations in primary and middle schools (i.e., early adolescence).

## 5 Conclusion

In conclusion, this study demonstrates that emotional reactivity is a significant risk factor for non-suicidal self-injury (NSSI) among adolescents, and that distress tolerance plays a central mediating role in this association. Higher emotional sensitivity, intensity, and persistence were linked to lower distress tolerance, which in turn increased adolescents’ likelihood of engaging in NSSI. The mediating pathway was evident primarily among females, highlighting potential gender differences in the emotional processes associated with self-injury. These findings enhance understanding of the emotional mechanisms underlying adolescent NSSI and underscore the importance of strengthening distress tolerance and emotion-regulation abilities in prevention and intervention efforts. Programs that cultivate emotional awareness, coping skills, and tolerance for negative affect may help reduce NSSI, particularly among adolescents with heightened emotional reactivity and among girls who are more vulnerable to distress-driven self-injury.

## Data Availability

The raw data supporting the conclusions of this article will be made available by the authors, without undue reservation.
